# The mutational load and a T-cell inflamed tumour phenotype identify ovarian cancer patients rendering tumour-reactive T cells from PD-1^+^ tumour-infiltrating lymphocytes

**DOI:** 10.1038/s41416-020-01218-4

**Published:** 2021-01-05

**Authors:** Diego Salas-Benito, Enrique Conde, Ibon Tamayo-Uria, Uxua Mancheño, Edurne Elizalde, David Garcia-Ros, Jose M. Aramendia, Juan C. Muruzabal, Julia Alcaide, Francisco Guillen-Grima, Jose A. Minguez, Jose Amores-Tirado, Antonio Gonzalez-Martin, Pablo Sarobe, Juan J. Lasarte, Mariano Ponz-Sarvise, Carlos E. De Andrea, Sandra Hervas-Stubbs

**Affiliations:** 1 Department of Medical Oncology, Clínica Universidad de Navarra, Pamplona, Spain; 2 Instituto de Investigación Sanitaria de Navarra (IdiSNA), Pamplona, Spain; 3 Program of Immunology and Immunotherapy, Center for Applied Medical Research (CIMA), University of Navarra, Pamplona, Spain; 4 Department of Pathology, Clínica Universidad de Navarra, Pamplona, Spain; 5 Department Pathology, Anatomy and Physiology, Universidad de Navarra, Pamplona, Spain; 6 Department of Gynecologic Oncology, Complejo Hospitalario de Navarra, Pamplona, Spain; 7 Department of Oncology, Hospital Costa del Sol, Marbella, Spain; 8 Department of Preventive Medicine, Clínica Universidad de Navarra, Pamplona, Spain; 9 Department of Obstetrics and Gynecology, Clínica Universidad de Navarra, Pamplona, Spain; 10 Department of Gynecology, Hospital Costa del Sol, Marbella, Spain; 11 GEICO Study Group, Madrid, Spain; 12 CIBERehd, Instituto de Salud Carlos III, Madrid, Spain; 13 Program of Solid Tumors, CIMA, University of Navarra, Pamplona, Spain; 14 Centro de Investigación Biomédica en Red de Oncología (CIBERONC), Madrid, Spain

**Keywords:** Tumour immunology, Immunotherapy

## Abstract

**Background:**

Adoptive immunotherapy with tumour-infiltrating lymphocytes (TIL) may benefit from the use of selective markers, such as PD-1, for tumour-specific T-cell enrichment, and the identification of predictive factors that help identify those patients capable of rendering tumour-reactive TILs. We have investigated this in ovarian cancer (OC) patients as candidates for TIL therapy implementation.

**Methods:**

PD-1^−^ and PD-1^+^ CD8 TILs were isolated from ovarian tumours and expanded cells were tested against autologous tumour cells. Baseline tumour samples were examined using flow cytometry, multiplexed immunofluorescence and Nanostring technology, for gene expression analyses, as well as a next-generation sequencing gene panel, for tumour mutational burden (TMB) calculation.

**Results:**

Tumour-reactive TILs were detected in half of patients and were exclusively present in cells derived from the PD-1^+^ fraction. Importantly, a high TIL density in the fresh tumour, the presence of CD137^+^ cells within the PD-1^+^CD8^+^ TIL subset and their location in the tumour epithelium, together with a baseline T-cell-inflamed genetic signature and/or a high TMB, are features that identify patients rendering tumour-reactive TIL products.

**Conclusion:**

We have demonstrated that PD-1 identifies ovarian tumour-specific CD8 TILs and has uncovered predictive factors that identify OC patients who are likely to render tumour-specific cells from PD-1^+^ TILs.

## Background

The adoptive transfer of tumour-infiltrating T lymphocytes (TILs) has shown clinical efficacy in patients with metastatic melanoma^1^ and cervical cancer.^2^ TIL therapy consists of the ex vivo expansion of T cells from tumour material and transfers back into the same patient after a lymphodepleting preparative regimen. Accumulating evidence supports ovarian cancer (OC) as a candidate for TIL therapy.^3,4^ However, attempts to treat OC patients with TILs have been generally disappointing.^5–7^

An important factor that determines the success of this therapy is the antitumour reactivity of the infused cellular product.^2,8^ During the TIL expansion process, there is an inter-clonal competition with different T-cell clones increasing or decreasing in frequency. Therefore, the more tumour-specific clones there are in the starting culture, the greater the chance of maintaining tumour-specific clones at an appreciable frequency in the final product. This may explain why the TIL therapy has yielded objective responses in cancers with high numbers of tumour-specific TILs, such as melanoma^9,10^ and virus-associated tumours,^2^ but not in cancers with lower numbers of tumour-specific TILs, such as OC.^5–7^

Surface markers, such as a programmed cell death protein 1 (PD-1) and CD137, have been proven to be able to identify melanoma-specific CD8 TILs.^11,12^ The CD8^+^CD137^+^ population is minority and largely confined to the CD8^+^PD-1^+^ TIL subset. For this reason, PD-1 has been proposed as the marker that more comprehensively identifies the repertoire of tumour-specific TILs.^11^ Recently, we have shown that the enrichment and separate amplification of PD-1^+^ CD8 TILs improves the antitumour efficacy of TIL therapy in mouse models of solid tumours.^13^ Our results were further confirmed by Jing et al. in haematological tumours.^14^

Overall, these findings have important implications since PD-1 may enable the isolation of rare tumour-specific lymphocytes and allow TIL therapy to be extended to less immunogenic tumours. Several pieces of evidence suggest that PD-1 may also demarcate tumour-specific CD8 T cells in ovarian tumours. Thus, the expression of PD-1 in intraepithelial CD8 TILs has been associated with a favourable prognosis in high-grade serous ovarian carcinomas.^15^ Prior studies have shown that NY-ESO-1-specific CD8^+^ TILs from OC patients expresses PD-1.^16^ In addition, ovarian tumours with deficient homologous recombination (HR) have a higher predicted neoantigen (NeoAg) load and infiltrating CD8 T cells in these tumours have increased PD-1 expression.^17^

Recently, investigation of potential biomarkers that may predict sensitivity to immunotherapy has become an area of active research. In the case of TIL therapy, a number of promising biomarkers have been discovered in the infused cell product that could predict clinical response.^18,19^ However, little is known about the factors in the original tumour that may help identify patients able to render tumour-reactive TIL products and, therefore, that might be eligible for this therapy.

In this study, we have investigated the role of PD-1 as a selective marker for the pre-enrichment, before expansion, of tumour-specific CD8 TILs in OC. Using multiple molecular and cellular analyses, we have also examined tumours at baseline to identify parameters that help us predict from which patients we can expect to obtain tumour-specific TILs.

## Methods

### Patients and tumour processing

We evaluated 10 chemotherapy-naive patients with different epithelial OC subtypes, including 7 high-grade serous carcinomas, 2 endometrioid carcinomas and 1 mucinous cystadenocarcinoma, at different cancer stages as described in Supplementary Table S1. The study was approved by the Institutional Review Boards of the Clinica Universidad de Navarra. Tumour fragments (*n* = 2–3, 0.7–1 cm^3^) were mechanically and enzymatically disrupted and the single-cell suspension was used to obtain tumour and non-tumour cell-enriched fractions (Supplementary Fig. S1), as detailed in Supplementary Methods. Both fractions were separately cryopreserved until further use.

### Flow cytometry (FC) analysis

Fresh tumour single-cell suspensions were incubated with Zombie NIR dye (Biolegend) and, subsequently, were stained with fluorochrome-conjugated monoclonal antibodies (mAbs) against EPCAM, CD45, CD3, CD4, CD8, PD-1 and CD137. Information on mAb clones and fluorochromes is found in Supplementary Methods. Cells were acquired in a FACSCanto-II cytometer (BD Biosciences, Franklin Lakes, NJ, USA) and analysed using FlowJo software (BD Biosciences).

### TIL isolation and expansion

Non-tumour cell-enriched fractions were thawed and rested overnight in T-cell media [1:1 mix of RPMI-1640-glutamax (Gibco) and AIMV (Gibco), supplemented with 5% heat-inactivated human serum (SIGMA), 12.5 mM HEPES, 100 U/ml penicillin, 100 μg/ml streptomycin and 10 μg/ml gentamicin (Gibco)]. The next day, cells were stained with fluorochrome-conjugated mAbs against CD8 and PD-1, and 7-amino-actinomycin D (dead cell marker), and were sorted into PD-1-negative (PD-1^−^) and PD-1 high (PD-1^hi^) CD8^+^ T cells using a FACSAria cell sorter (BD Biosciences). Isolated cells were separately expanded by Rapid expansion protocol (REP) in T-cell media containing soluble anti-CD3 mAb (OKT3) (30 ng/ml, Biolegend), human IL-2 (3000 IU/ml, Proleukin) and 3 × 10^7^ irradiated peripheral blood leucocytes pooled from 3 different donors. Detailed information is found in Supplementary Methods and Supplementary Table S2. After 12–15 days of expansion, T cells were cryopreserved until further analysis.

### TIL reactivity assessment

Expanded CD8 TILs and autologous tumour-enriched cells were thawed and separately rested overnight in T-cell media without IL-2. The next day, TILs (5 × 10^4^ cells/well) were co-cultured either alone or with target cells [autologous tumour-enriched cells or the H929 plasmacytoma cell line (ATCC), as unrelated tumour cells] (10^5^ cells/well) in ELIIP plates (Millipore) coated with purified anti-human IFN-γ mAb (Mabtech), in the presence or absence of HLA-I blocking mAb (W6/32, Bio-x-cell). Thirty-six hours later, plates were developed as described in Supplementary Methods. The results were expressed as the number of IFN-γ^+^ spots. When autologous tumour-enriched cells were available, TIL reactivity was confirmed by assessment of CD137. Briefly, TILs (1 × 10^5^ cells/well) were cultured, either alone or with target cells (0.5–1 × 10^5^ cells/well), in T-cell media, and 24 h later, cells were analysed for CD137 expression by FC.

### Multiplex immunofluorescence staining and analysis

Multiplex immunofluorescence (IF) staining, validation and analysis are detailed in Supplementary Methods and Supplementary Figs. S2 and S3. Briefly, 4-μm sections of formalin-fixed paraffin-embedded (FFPE) tissue samples were deparaffinised and antigen retrieval was performed using DAKO PT-Link heat-induced antigen retrieval with a low- (pH 6) or high-pH (pH 9) solution (DAKO). Samples were stained with mAbs targeting cytokeratin (CK), CD4, CD8, FOXP3, PD-1 and CD137 followed by TSA visualisation with fluorophores Opal 520, Opal 540, Opal 570, Opal 620, Opal 650 and Opal 690 (Akoya Biosciences), as described earlier.^20^ Each tissue section was put through several sequential rounds of antibody staining. In the seventh round, nuclei were counterstained with spectral DAPI (Akoya Biosciences) and sections mounted with Faramount Aqueous Mounting Medium (Dako). Multiplexed immunofluorescence slides were scanned on a Vectra-Polaris Automated Quantitative Pathology Imaging System (Akoya Biosciences). Tissue imaging, spectral unmixing and phenotyping were performed using inForm software (version 2.4.8, Akoya Biosciences), as described previously.^20^

### Somatic mutation estimation

The mutational load was estimated using the Trusight Tumour 170 panel (TST170) from Illumina. FFPE-tumour DNA extraction, library preparation and sequencing (NextSeq 500 using high-output cartridge and v2 chemistry) were executed by Macrogen (Korea) according to Illumina’s instructions. Sequence alignment and variant calling were performed using the TST170 BaseSpace application. Variant annotation was performed using Annovar (dbSNP150, 1000G, gnomAD and COSMIC). Positions with depth <100, variant call quality <40, NC (fraction of bases that were uncalled or with base call quality below the minimum threshold) ≥0.03 (for SNVs), strandbias score > −80 and variant allele frequency (VAF) <0.05 were ignored. Germline polymorphism filtering was performed by filtering variant allele frequency (VAF) between 0.4–0.6 and > 0.8. Polymorphisms were excluded if their minor allele frequencies (MAF) in European non-finnish (ENF) population (the ethnic group of our patients) according to gnomAD were > 0. Mutations with MAF(ENF) = 0 or not found in gnomad were also not found in 1000G database. All putative somatic SNVs and INDELs were carefully checked by manual inspection of the sequenced reads using the Integrative Genome Browser (IGV). About 100% of SNVs were “truly SNVs”, whereas 60% of identified somatic indels were “False positive”. Due to the high rate of false-positive Indels and the lower proportion of these variants in OC (~3% of the number of indels + SNVs),^21^ only SNVs were considered for the mutational load estimation. Tumour mutational burden (TMB) was calculated as the number of somatic SNVs per 0.524 megabase pairs (the targeted genomic regions of TST170). For more detailed information, see Supplementary Methods and Supplementary Tables S3–S11.

### NanoString-based gene expression profiling

FFPE-tumour RNA was extracted using the RNeasy FFPE kit (QIAGEN). nCounter PanCancerImmune Profiling panel analysis (Nanostring) was performed by IMIBIC (Spain) following the manufacturer’s instructions. Analyses of expression were performed using nSolver Analysis Software (v 4.0), with normalisation utilising positive and negative control probes, as well as the most stable housekeeping (HK) genes across samples. *p* values < 0.05 were considered to identify differentially expressed genes (DEG). Volcano plots and unbiased clustering of DEG were generated using R (v3.5.3). For analysis of immune signatures, after HK normalisation, a log10 transformation was applied, and the signature score was calculated by averaging the expression level of those genes included in the IFN-γ signature, Expanded immune signature and T-cell inflamed signature.^22^ For more information, see Supplementary Methods.

### Statistical analysis

The statistical tests used are detailed in each figure legend. For detailed information, see Supplementary Methods.

## Results

### TILs in fresh ovarian tumours display variable expression of PD-1 and CD137

**Fig. 1:**
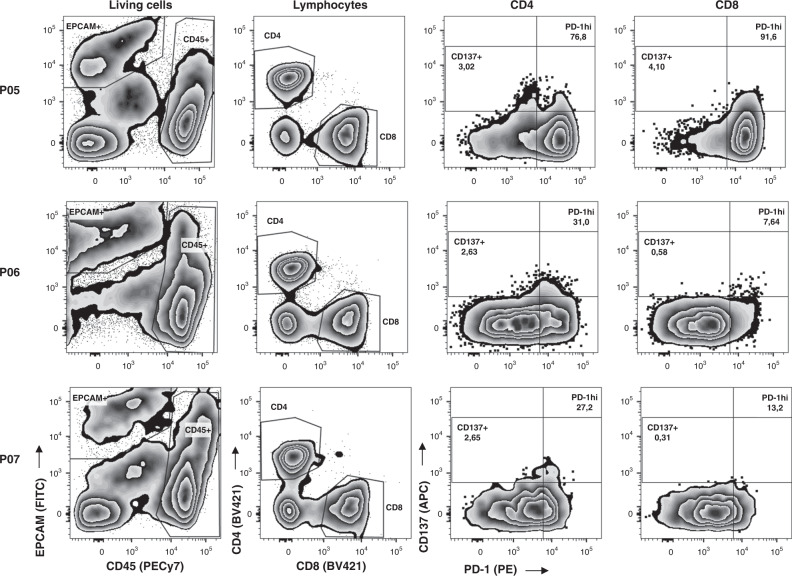
PD-1 and CD137 expression in CD4^+^ and CD8^+^ TILs in tumour samples from OC patients. Tumour single-cell suspensions were analysed by FC as detailed in ‘Methods’. Gating strategy is described in Supplementary Fig. S4. The figure shows three representative patients (P05, P06 and P07). Names at the top indicate the parental population. Numbers indicate the percentage of gated cells with respect to the parental population.

Single-cell suspensions of fresh human ovarian tumours comprised both CD45^+^ cells and EpCAM^+^ cancer cells (Fig. 1 and Supplementary Fig. S4). The percentages of CD4^+^ and CD8^+^ cells within the CD45^+^ population (Supplementary Fig. S5A) ranged from 3.6 to 36.1% and from 5.9 to 31.6%, respectively. CD4^+^ and CD8^+^ TILs expressed PD-1 at variable levels (range of 1.73–72.7% and of 0.1–88.6% for CD4^+^ and CD8^+^ cells, respectively) (Fig. 1 and Supplementary Fig. S5B). Expression of CD137 was much lower than that of PD-1 and confined to the PD-1^+^ subsets. Interestingly, CD137 in CD8^+^ TILs was almost exclusively expressed on PD-1^hi^ cells. The level of CD137 expression within the PD-1^+^CD4^+^ and the PD-1^+^CD8^+^ subset varied among patients (Supplementary Fig. S5C).

### CD8 TIL reactivity against autologous tumour was confined to the PD-1^high^ compartment

**Fig. 2:**
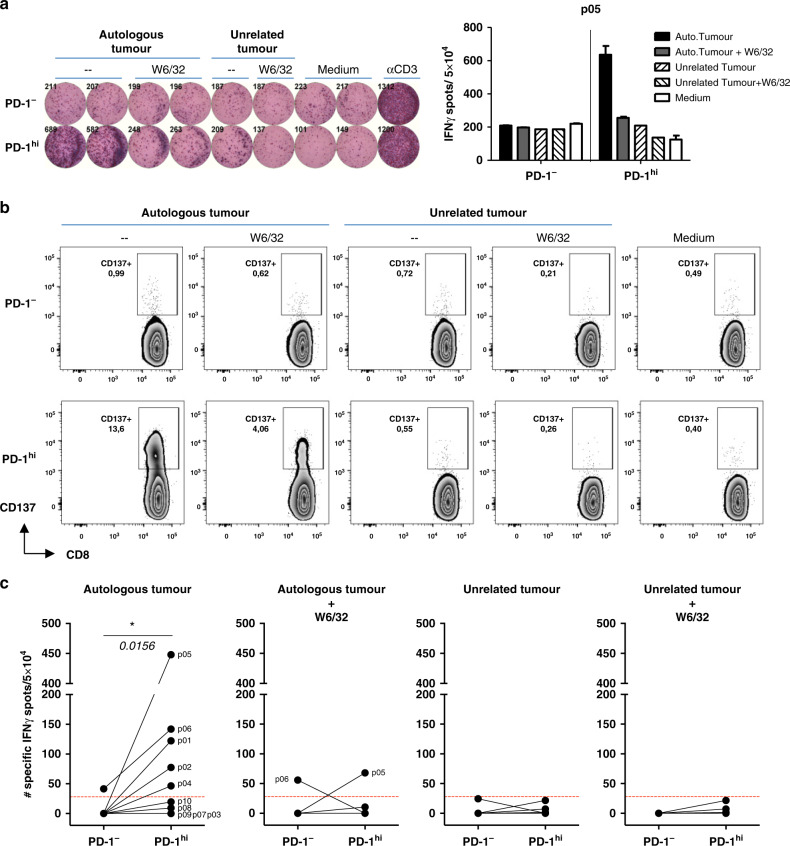
Cells derived from the PD-1^hi^ CD8 TIL subset recognise autologous tumour cells. Cells expanded from the PD-1^−^ or the PD-1^hi^ CD8 TIL subset isolated from different patients were co-cultured with the respective autologous tumour cells, or with unrelated tumour cells (H929), in the presence or absence of HLA-I blocking antibody (W6/32), and tumour recognition was assessed by measuring IFN-γ release by ELISPOT (**a**, **c**) and the frequency of CD137^+^ cells by FC (**b**). **a**, **b** Antitumour reactivity of cells derived from PD-1^−^ and PD-1^hi^ CD8^+^ TILs from patient P05. **a** Left: picture of the ELISPOT plate. Plate-bound anti-CD3 mAb was used as a positive control. Right: the graph shows the number of spots per 5 × 10^4^ cells (mean ± SD). **b** Dot plots display the frequency of CD137^+^ cells in PD-1^−^ (up) and PD-1^hi^ (down) derived CD8 TILs 24 h after co-culture with target cells. Cells are gated on blastic CD45^+^CD3^+^CD8^+^cells. **c** Antitumour reactivity of cells derived from PD-1^−^ and PD-1^hi^ CD8^+^ TILs from each patient. The number of target-specific IFN-γ spots was determined by calculating the difference between the number of spots generated in the presence of target cells (autologous tumour or nonrelated tumour) and twice the number of spots generated in the absence of targets cells (medium). Reactivities >30 specific IFN-γ spots were considered positive (horizontal dotted line). Each line matches the PD-1^−^ and PD-1^hi^ TIL products derived from the same patient. Mean ± SEM. *P ≤ 0.05. Wilcoxon matched-pair signed-rank test, two-tailed, 95% confidence level.

To determine if PD-1 may enrich tumour-specific T cells in OC, we isolated CD8 TILs with extreme expression of PD-1, namely PD-1^−^ and PD-1^hi^ cells, from 10 resected ovarian tumours and expanded them separately. The number of isolated PD-1^−^ and PD-1^hi^ CD8 TILs varied among patients. Both subsets expanded efficiently (Supplementary Table S2). Next, we tested the ability of the expanded cells (also referred to as TIL products) to recognise the autologous tumour using the enriched tumour cell fraction obtained from enzyme-digested tumours. TIL products were cultured alone or together with autologous tumour cells or unrelated tumour cells (H929) in the presence or absence of HLA-I blockade. Figure 2a, b shows data from patient P05. Notably, cells derived from the PD-1^hi^ CD8 TIL subset, but not from the negative counterparts, were tumour-reactive (TR) cells, as determined by IFN-γ secretion (Fig. 2a) and CD137 upregulation (Fig. 2b). We found TR TILs in 5 out of 10 patients (Fig. 2c and Supplementary Fig. S6). Remarkably, the antitumour reactivity was harboured by cells derived from the PD-1^hi^ compartment, as deduced by IFN-γ ELISPOT. Recognition of autologous tumour by PD-1^hi^-derived cells was HLA-I-restricted (Fig. 2 and Supplementary Fig. S6). Only PD-1^−^- derived cells from patient P06 were able to recognise autologous tumour, but this recognition was not HLA-I-restricted (Fig. 2c and Supplementary Fig. S6). Recognition was tumour specific since TILs did not respond to unrelated H929 tumour cells (Fig. 2 and Supplementary Fig. S6). Our data indicate that, although only PD-1^+^-derived T cells were able to recognise autologous tumour cells, the ability of PD-1-selected cells to render TR TIL products varied among patients. Accordingly, patients were divided into two groups: patients with TR TILs (P01, P02, P04, P05 and P06) and patients with non-tumour-reactive (NTR) TILs (P03, P07, P08, P09 and P10).

### Detection of CD137^+^ cells within the PD-1^hi^CD8^+^ TIL subset in the fresh tumour correlates with the antitumour reactivity of the final TIL product

**Fig. 3:**
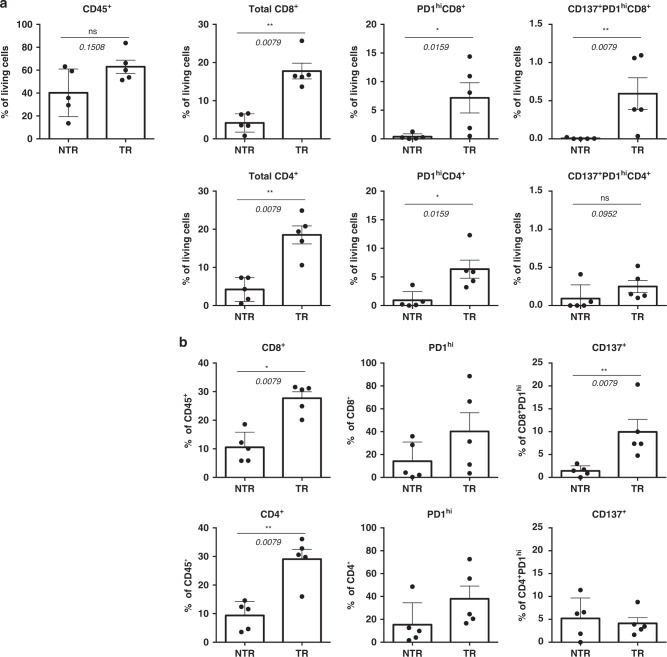
Detection of CD137^+^ cells within the PD-1^hi^CD8^+^ TIL subset in the fresh tumour correlates with antitumour reactivity of the final TIL product. The phenotypic traits of CD4 and CD8 TILs in the fresh tumour (Supplementary Fig. S5) from patients who rendered TR TILs were compared with those from patients with NTR TIL products (as defined in Fig. 3). **a** Frequency of the indicated subsets in living cells (Zombi NIR^−^). **b** Frequency of CD8^+^, CD4^+^, PD-1^hi^ and CD137^+^ cells in the respective parental population. Each dot represents 1 patient’s sample. Mean ± SEM. *P ≤ 0.05; **P ≤ 0.01, Mann–Whitney test, two-tailed, 95% confidence level.

We retrospectively analysed FC data from tumour infiltrates to find any feature that distinguished patients from the TR and NTR groups. No statistically significant differences were found in the percentage of CD45^+^ cells between both groups (Fig. 3a). Notably, those patients delivering TR TILs presented a significantly higher frequency of CD8^+^ and CD4^+^ lymphocytes in the fresh tumour (Fig. 3a, b). These patients also showed a higher proportion of PD-1^+^CD8^+^ and PD-1^+^CD4^+^ TILs (Fig. 3a). Interestingly, while no difference was found in the percentage of CD137^+^PD-1^+^CD4^+^ cells, those patients who rendered TR TILs showed a higher proportion of CD137^+^PD-1^+^CD8^+^ TILs (Fig. 3a). These patients also presented a higher frequency of CD137^+^ cells within the PD-1^hi^CD8^+^ population (Fig. 3b), the cell subset sorted for further expansion. No differences were found either in the percentage of PD-1^+^ cells within the CD8^+^ and CD4^+^ TIL compartment, or in the percentage of CD137^+^ cells within the PD-1^hi^CD4^+^ population (Fig. 3b).

### Patients with TR TIL products exhibited a higher percentage of CD137^+^PD-1^+^CD8^+^ TILs in the tumour epithelium

**Fig. 4:**
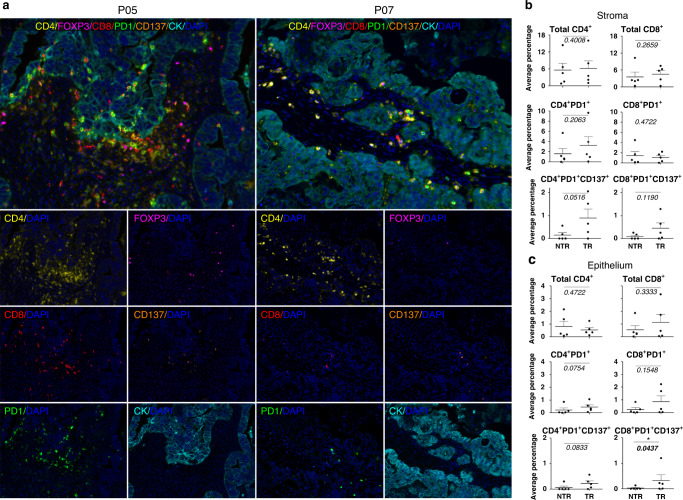
Multiplex immunofluorescence assessment of different TIL subsets in the tumour microenvironment. Tumours were analysed by multiplexed IF using mAbs targeting CK (cyan), CD4 (yellow), CD8 (red), PD-1 (green), CD137 (orange) and Foxp3 (magenta) molecules. **a** Representative fluorescence images corresponding to patient P05 (TR group) and patient 07 (NTR group). Immunofluorescent multiplexing images were scanned with the Akoya Biosciences’ multispectral image platform. Figures at the top compile the entire staining (merge). **b**, **c** Total CD8^+^ and CD4^+^ cells, PD-1^+^CD8^+^ and PD-1^+^CD4^+^ cells (regardless of whether or not they express CD137) and CD137^+^PD-1^+^CD8^+^ and CD137^+^PD-1^+^CD4^+^ cells were quantified using multiplexed quantitative analysis. In order to evaluate TIL subpopulations, first the percentage of each cell population was calculated from the total number of cells in 20 random fields (representing in total 6.95 mm^2^ of tumour area for each case of ovarian cancer). Then, the average of the percentage of all fields was calculated. This evaluation was done in both the stroma (**b**) and epithelium (**c**) compartments. The CD137^+^PD-1^+^CD8^+^ cell population represented a very small subpopulation inside the tumour (nearly null for few patients). Patients who rendered TR TILs were compared with those with NTR TILs. No significant differences were found in the percentage of Foxp3^+^ TILs, neither in the epithelium nor in the stroma, between both groups (data not shown). Each dot represents 1 patient. Mean ± SEM. Mann–Whitney test, one-tailed, 90% confidence level, Monte Carlo exact *p*-value estimation.

We subsequently analysed tumours by multiplexed IF (Fig. 4). Representative fluorescence images from patients P05 (TR group) and P07 (NTR group) in Fig. 4a show the presence of CD4^+^ and CD8^+^ T cells in the tumour infiltrate. CD4^+^ and CD8^+^ TILs were more prominent in the tumour stroma than in the tumour epithelium (Fig. 4b, c). PD-1, CD137 and forkhead box P3 (Foxp3) expression was also detected in the tumour infiltrate, with PD-1 being the most notable marker (Fig. 4a).

Since FC analyses showed that patients who rendered TR TILs had a higher percentage of CD137^+^PD-1^+^CD8^+^ cells in the tumour, we also analysed this phenotype by multiplexed quantitative IF. Total CD4^+^ and CD8^+^ TILs, PD-1^+^CD4^+^ and PD-1^+^CD8^+^ TILs (regardless of whether or not they expressed CD137) and CD137^+^PD-1^+^CD4^+^ and CD137^+^PD-1^+^CD8^+^ TIL subsets were quantified (Fig. 4b, c). Multiplexed quantitative IF showed that the percentage of CD137^+^PD-1^+^CD8^+^ cells among the total number of cells was very small. A relatively large tumour area was evaluated (6.95 mm^2^ of tumour area); thus, CD137^+^PD-1^+^CD8^+^ cells represented a very small cell population in the tumour. Patients with TR TIL products exhibited a slightly higher, but statistically significant, percentage of CD137^+^PD-1^+^CD8^+^ TILs in the tumour epithelium (Fig. 4c).

### Antitumour reactivity of the final TIL products was associated with higher mutational load

**Fig. 5:**
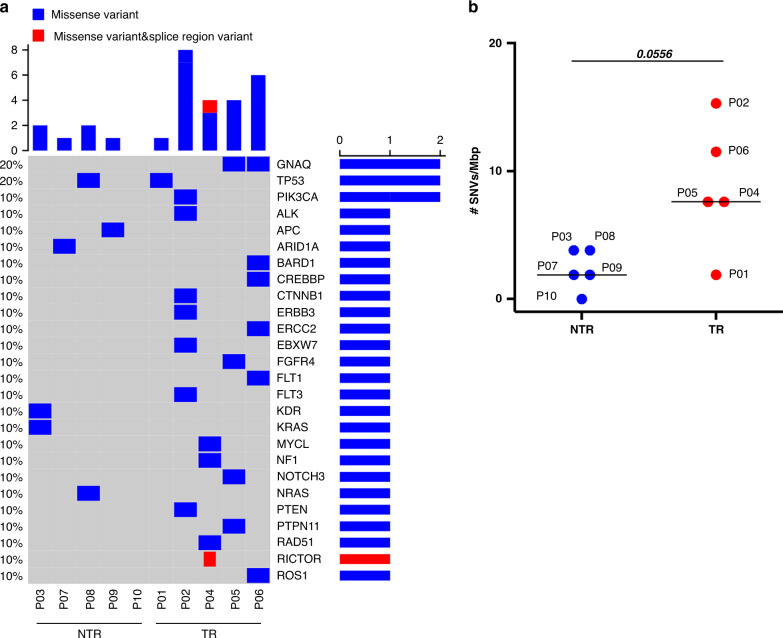
Patients rendering TR TILs exhibited higher predicted mutational load than those with NTR TILs. **a** Oncoprint displaying non-synonymous somatic SNVs in 26 cancer genes across studied patients (Supplementary Table S4). The digits on the left indicate the percentage of patients who presented mutation in the indicated gene. The bars on the right show the total number of mutations in each gene. **b** Estimation of SNV load with TST170 panel. Patients who rendered TR TILs were compared with those with NTR TILs. Each dot represents 1 patient. Mann–Whitney test, two-tailed, 95% confidence level.

Somatic mutations may potentially generate neoAgs capable of eliciting a highly potent and tumour-specific immune response. In order to determine if the number of mutations was related to the antitumour reactivity of the final TIL products, we estimated the mutational load of our patients using the TST170 panel as detailed in Supplementary Methods. Of the 2068 variants identified (Supplementary Table S3), 126 were SNVs and only 29 met the criteria for being somatic (Supplementary Table S4 and S5). Mutations were distributed along 26 genes (Fig. 5a). The median of SNVs/Mbs was 3.8, ranging from 0 to 15.3 (Supplementary Table S5). Remarkably, those patients with the highest mutational load (P02 and P06) belonged to the group with TR TILs (Supplementary Table S5). Interestingly, the SNV load of the TR group was borderline significantly higher than that of the NTR group (*p* = 0.0556) (Fig. 5b).

We also analysed mutations in genes from the DNA damage repair (DDR) system. Defects in these pathways may accumulate an enormous number of mutations due to genome instability during the carcinogenic process and may account for the different mutational load found in our patients. Twenty-eight genes out of about 120 genes involved in direct DDR^23^ were contained in the TST170 panel (Supplementary Table S9–S11). A total of 53 variants were detected (5.3 ± 1.9) affecting 18 genes (Supplementary Table S9). To identify disturbing mutations affecting these genes, the degree of certainty of pathogenicity was assessed in different databases, as detailed in Supplementary Methods. Only 3 variants met the criteria to be putatively clinically relevant mutations (Supplementary Table S9). Notably, these variants were solely found in those patients with the highest mutational load. Indeed, P02 exhibited clinically relevant mutations in the ATM and FBXW7 genes, whereas P06 presented a likely pathogenic mutation in ERCC2.

### Immune activation gene signature at the tumour site identified patients rendering TR TILs

**Fig. 6:**
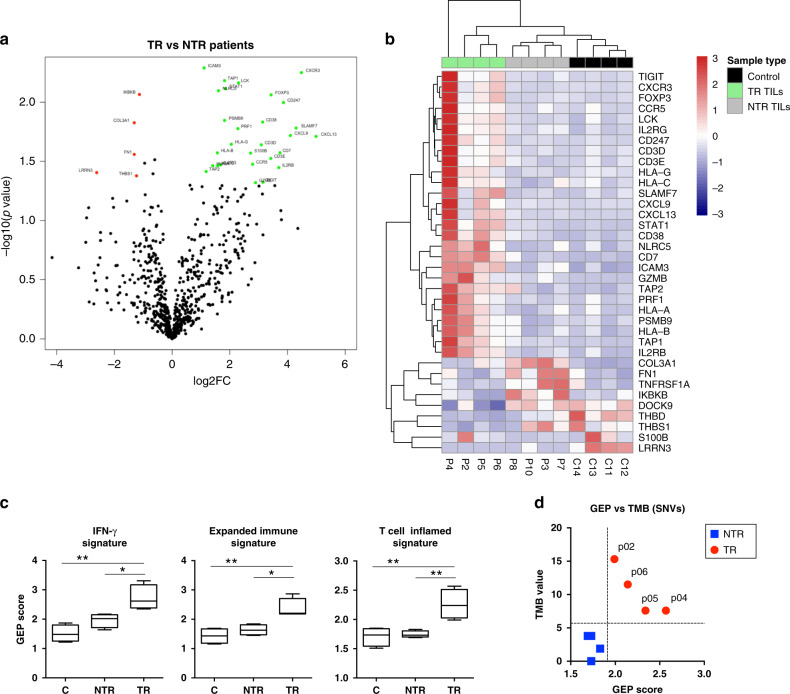
Immune activation gene signature in baseline tumour samples distinguishes patients rendering TR TILs. The expression of 770 immune-related genes was profiled using the PanCancerImmune Profiling Panel. Only FFPE-tumour RNA samples that met the quality control requirements [percentage of 300 nucleotide-long RNA fragments >50% and high purity] were included in the study. They included RNAs from 8 patients (P02, P04, P05 and P06, from TR group, and P03, P07, P08 and P10, from NTR group) and 4 ovaries with non-malignant disease (C11–C14). **a** Volcano plot for DEG between the TR and NTR groups. The coloured circles indicate significant DEG (*p* < 0.05) with fold change >2 (green) or <0.5 (red). **b** Heatmap showing the assortment of patients and healthy ovaries (based on the top 36 DEG between TR and NTR groups). Rows represent genes and columns patients and controls. Gene expression was standardised by the mean of the samples. The rows and columns have been grouped using unsupervised hierarchical clustering. **c** Gene expression signatures associated with TR TIL products. GEP score was calculated by averaging the expression level of the genes included in the *IFN-γ signature*, *Expanded immune signature* and *T-cell inflamed signature*. **d** GEP score was plotted against the TMB value in each patient. *P ≤ 0.05; **P ≤ 0.01. Mean ± SEM. Mann–Whitney test, two-tailed, 95% confidence level.

Whereas high values of TMB seemed to be related to the antitumour reactivity of the TIL products, the fact that patients with relatively low TMB values also gave TR TILs suggests that other mechanisms independent of neoantigenicity might also account for the capability of rendering TR TILs. Gene signatures of activated T cells have been shown to predict response to anti-PD-1 therapy.^22^ In order to determine if the gene expression profile (GEP) may also predict patients capable of eliciting TR TIL products, we analysed the expression of 770 immune-related genes in 8/10 patients with FFPE-tumour RNA available. Four ovaries with non-malignant disease were included as a control. Transcriptome analysis revealed a set of 36 DEG between the TR and NTR groups (Fig. 6a). Unsupervised hierarchical clustering clearly separated patients from TR and NTR groups, as well as the control ovaries (Fig. 6b).

Gene Ontology analysis identified biological processes and pathways relevant to the antitumour response that were significantly overrepresented in tumours from the TR group (Supplementary Fig. S7A and B). Interestingly, high baseline expression of genes involved in antigen processing/presentation [PSMB9, TAP1, TAP2, HLA-I (A, B and C) and NLRC5], T-cell activation [CD247 (CD3z), CD3E, CD3D, LCK, CD7, CD38 and ICAM3], proliferation/differentiation of cytotoxic T cells (IL2RB and IL2RG), cytotoxic activity (PRF1 and GZMB), IFN‑γ signalling pathway (STAT1) and chemoattraction (CXCL9, CXCL13, CXCR3 and CCR5), as well as inhibitory mechanisms (HLA-G, FOXP3 and TIGIT), was associated with TR TIL products (Fig. 6b). In addition, patients with TR TIL products exhibited a higher expression of the TNFRSF9 (CD137) transcript that was close to statistical significance (Supplementary Fig. S7C). In contrast, higher expression of genes involved in wound healing (COL3A1, FN1, THBD, THBS1 and TNFRSF1A), mesenchymal transition and cell adhesion (COL3A1 and FN1), angiogenesis and blood vessel development (COL3A1 and THBS1), invasiveness (DOCK9 and THBS1) as well as NF-Κb [IKBKB and TNFRSF1A (TNFR1)] and Ras-MAPK (LRRN3) pathways, was associated with NTR TIL products.

Recently, three immune-related gene expression signatures that predict clinical response to PD-1 blockade across multiple cancer types have been identified: the IFN-γ signature, the Expanded immune gene signature and the T-cell inflamed signature.^22^ Interestingly, patients rendering TR TIL products exhibited significantly higher scores for the three mentioned signatures, in particular for the T-cell inflamed GEP (Fig. 6c).

Although the correlation between TMB and GEP was low, both parameters stratified TR and NTR patients (Fig. 6d). Therefore, patients with high values of either TMB (P02, P05 and P06) or GEP (P04) rendered TR TIL products, whereas those with low values of both biomarkers (P03, P07, P08 and P10) did not.

Finally, we also analysed the expression of cancer testis antigens (CTA) included in the panel. No differentially expressed CTA genes were detected between the TR and the NTR groups (Supplementary Fig. S7C). Curiously, patient P05, who rendered highly TR TILs (Fig. 2), stood out for expressing a large number of CTA genes.

## Discussion

Adoptive immunotherapy using TILs in patients with melanoma^1^ and cervical^2^ cancer frequently results in durable complete responses. However, in OC patients, responses to TIL therapy have been less dramatic^5–7^ due in part to technical difficulties in identifying and expanding the TR TIL subset. In an effort to improve TIL therapy in OC, in this study, we have investigated the ability of PD-1 marker for ovarian tumour-specific T-cell enrichment. We have found that autologous tumour-specific T cells were exclusively present in cells derived from PD-1^+^CD8^+^ TILs, but not from their PD-1^−^ counterparts. These findings provide a method to preselect ovarian tumour-specific T cells directly from tumour infiltrates, without the need for complex immunological screening technologies that require antigen prediction/identification to evaluate T cells. Recently, it has been shown that PD-1 also allows the identification of tumour-specific T cells in  mismatch repair-proficient gastrointestinal cancer patients.^24^ This finding together with our results indicates that the utility of PD-1 to select for tumour-specific T cells is not restricted to highly immune-reactive tumours.^11,13,14,25^

However, only in 5 out of 10 patients studied, the PD-1^+^-derived cells were able to recognise autologous tumour. Interestingly, those patients with TR TIL products exhibited a higher frequency of CD137^+^ cells within the sorted PD-1^+^CD8^+^ population. Our findings reopen the debate on which marker, PD-1 or CD137, best identifies the repertoire of tumour-specific T lymphocytes.^11,12^ In the only patient studied, Ye et al. found that PD-1^+^CD137^+^ cells, but not the PD-1^+^CD137^−^ subset, harboured tumour-specific T cells in OC.^12^ These results appear to contradict Rosenberg group’s findings in melanoma,^11^ which showed that both populations comprised tumour-specific CD8 TILs. CD137 expression is an indication of ongoing or recent activation of T cells,^26^ in contrast to PD-1 expression, which is maintained long after antigen recognition. On the other hand, the PD-1^+^ TIL population is heterogeneous and houses bystander and tumour-specific T cells,^27^ some of which may be recently activated cells (PD-1^+^CD137^+^), whereas the large majority are cells activated long ago (PD-1^+^CD137^−^). The ratio between bystander and tumour-specific TILs varies between tumours,^28^ and this could explain why in OC, with smaller numbers of tumour-specific lymphocytes in comparison to melanoma,^11^ only CD137^+^ cells^12^ or PD-1^+^ cells containing a high proportion of CD137^+^ cells (our study) were able to render TR TILs. A more exhausted state of PD-1^+^ tumour-specific TILs in OC may also account for the difficulty of obtaining TR TIL products from this population. Patient-related factors may also be implicated. Thus, similar to what we have found in OC, the ability of PD-1 to enrich for tumour-specific TILs in melanoma patients has also been shown to be inconsistent.^25^

Therefore, the use of PD-1 as a single marker to isolate tumour-specific cells appears to be controversial. On the other hand, the expression of CD137 is transitory. Consequently, a large proportion of tumour-specific T cells may be missed in the CD137^−^ fraction. A recent study in colorectal and lung cancer has shown that CD39 accurately distinguishes between tumour-specific (PD-1^+^CD39^+^) and bystander (PD-1^+^CD39^−^) TILs.^27^ In another study, co-expression of PD-1 with markers of tissue-resident memory cells, such as CD39 and CD103, seemed to identify tumour-specific TILs.^29^ Presumably, combinations of PD-1 with other markers would be necessary to accurately identify the full repertoire of tumour-specific TILs, especially in tumours in which these cells are rare.

The finding that those patients rendering TR TILs had a higher frequency of CD137^+^PD-1^+^CD8^+^ TILs located in the tumour epithelium, suggests that their tumours may have special features that have enabled the activation of T cells, e.g., the presence of relevant antigens or/and an immune-permissive tumour microenvironment (TME). TMB provides an indirect assessment of tumour antigenicity because a high level of mutations will increase the likelihood of neoAg generation. The TMB values in our patients, as estimated using the TST170 panel, ranged from 0 to 15.3 SNVs/mpb. These values were within the range of those found for OC using whole-exome sequencing (WES), although the median values (3.8 SNVs/Mbp) were higher than those estimated with this method (~1.5 mutations/Mb).^30,31^ The use of a gene panel enriched in known cancer genes, which are more likely to be mutated in tumours, often leads to TMB overestimation.^32^ The precise estimation of the mutational load may also have been affected by the low genomic coverage of the TST170 panel (0.53 Mbp), a problem that most seriously affects tumours with low/moderate TMB levels, as is the case of OC.^33,34^ However, previous studies have shown that the numbers of mutations detected with the TST170 panel strongly correlate (Pearson correlation 0.97) with those observed with the gold standard WES.^34^ In addition, exploratory analyses demonstrated that the TST170 panel-based TMB discriminates between immunotherapy responders and non-responders.^35,36^ Therefore, although the panel used cannot provide an accurate estimation of the mutational load, it can still be valid to distinguish between patients. Remarkably, those patients with the highest SNV numbers (P02 and P06) belonged to the TR group. Although our data need to be confirmed in a larger cohort of patients using a higher panel size, they suggest that, in some patients at least, the existence of possible neoAgs might account for the capability of rendering TR TILs.

Several intrinsic mechanisms, including deficiencies in DDR genes, have been implicated in contributing to neoAg load.^37^ Interestingly, patient P02 showed deleterious mutations in ATM and FBXW7 genes, involved in HR and non-homologous end-joining pathways, respectively, whereas patient P06 presented a likely pathogenic mutation in ERCC2, entailed in the nucleotide excision repair pathway. ATM gene defects strongly correlate with spontaneous TIL infiltration in breast cancer and OC.^38^ Recently, it has been shown that bladder cancer patients with mutated ATM had better benefits from immune checkpoint inhibitor (ICI) therapy.^39^ Inactivation of FBXW7 causes genomic instability.^40^ Importantly, mutations in ATM and FBXW7 genes correlate with higher TMB levels in several cancer types, including OC.^41–47^ Mutated ERCC2 has also been associated with high TMB values.^41^ Although our findings require further investigation, they suggest that deleterious alterations in DDR genes may influence the competence to render TR TILs by leading to an increased TMB, and subsequently increased number of neoAgs.

The finding that some patients from the TR group showed relatively low mutational load suggests that mechanisms independent of neoantigenicity might also account for the capability of rendering TR TILs. The expression profile of immune-related genes reflects the nature of the TME, which is also an important feature of cancer immunobiology. Interestingly, tumours from patients with TR TIL products showed a high expression of genes involved in antigen processing/presentation, T-cell activation, proliferation/differentiation of T cells, cytotoxic activity, IFNγ-signalling, chemoattraction as well as mechanisms to regulate T-cell response. This GEP is characteristic of immune-reactive tumours with prolonged survival and response to ICIs.^48,49^ A similar GEP has also been associated with high tumour T-cell infiltration in OC patients.^50,51^ In line with the transcriptomic data, FC analysis of tumour infiltrates revealed a significantly higher frequency of CD8 and CD4 T cells in those patients who rendered TR TILs. Likewise, a high expression of genes involved in wound healing, mesenchymal transition, extracellular matrix remodelling, angiogenesis and invasiveness, was associated with the lack of antitumour reactivity of the final TIL product. The concurrent overexpression of genes involved in these processes has been associated with the presence of immunosuppressive TME,^52^ T-cell dysfunctionality^53^ and resistance to anti-PD-1 therapy.^48^

Three multigene expression signatures (IFN-γ, expanded immune and T-cell-inflamed signature) have been associated with clinical benefit from anti-PD-1 treatment in several cancer types.^22^ Importantly, patients rendering TR TIL products exhibited significantly higher scores for the three mentioned signatures, in particular for the T-cell inflamed GEP. These findings suggest that GEP may represent a useful tool to identify patients who are likely to render TR TILs and, ultimately, who may benefit from TIL therapy.

Interestingly, while the TMB values did not correlate with the GEP score, both parameters stratified TR and NTR groups. Thereby, patients with high values of either TMB and/or GEP rendered TR TIL products, whereas those with low values of both TMB and GEP did not. Our data show some similarities with the predictive value of TMB and GEP to anti-PD-1 therapy across different tumour types.^54,55^ The ability of these two parameters to reflect overlapping but not always coincident aspects of tumour immunology may explain their lack of correlation.

The fact that patients with relatively low TMB rendered TR TILs suggests that the quality of neoAgs, but not the quantity, or the presence of relevant shared tumour-associated antigens, may have determined the antitumour reactivity of the final TIL product. In the case of OC, several groups have identified TILs specific for private^56^ and hot-spot mutations,^57^ as well as for CTA.^4,16^ Interestingly, the patient who in our study stood out for rendering highly TR TILs (P05), exhibited a high expression of very potent CTA, such as NY-ESO-1, MAGEC2 and TPTE. In the future, it will be interesting to determine what type of antigens account for the antitumour reactivity of TIL products in OC.

The present study has notable limitations, namely the low number of patients and the inclusion of distinct histological subtypes of epithelial OC. Although our findings warrant additional investigation and need to be validated in larger-scale studies, they provide a rationale for further exploring the utility of biomarkers to (i) identify and selectively expand tumour-specific TILs, and (ii) serve as guides for selecting patients eligible for TIL therapy. In order to increase the tumour reactivity of the final TIL product, other selective markers may be considered together with PD-1 to distinguish tumour-specific from bystander T cells. The combination of several parameters, such as TIL density and the presence of intraepithelial CD137^+^ cells in the fresh tumour together with the GEP and the TMB, could guide future steps to defining an extensive predictive biomarker panel to identify patients eligible for TIL therapy. The use of such a predictive biomarker panel together with newer ways to select tumour-specific TILs, will help to extend this cell therapy to less immune-reactive tumours, such as OC.

## Supplementary information

Supplementary Methods, Supplementary Figures and Supplementary Tables S1 and S2

Supplementary Table S3

## Data Availability

All data generated or analysed during this study are included in this published article and its Supplementary files.
