# Supplementary material for: The mutational load and a T-cell inflamed tumour phenotype identify ovarian cancer patients rendering tumour-reactive T cells from PD-1+ tumour-infiltrating lymphocytes
Source: Br J Cancer. 2021 Jan 5;124(6):1138–49. doi: 10.1038/s41416-020-01218-4 (PMC7961070; doi:10.1038/s41416-020-01218-4)
Supplement: Supplementary file 1 — Supplementary Methods, Supplementary Figures and Supplementary Tables S1 and S2 [file 41416_2020_1218_MOESM1_ESM.pdf]

SUPPLEMENTARY METHODS

Patients and tumour processing

We evaluated 10 chemotherapy-naïve patients with different OC subtypes and at different cancer stages as described in **Supplementary Table S1**. The study was approved by the Institutional Review Boards of the Clinica Universidad de Navarra (Spain). After surgery, the fresh tumour underwent sterile dissection in the Anatomic Pathology Facility (Clinica Universidad de Navarra). A representative tumour sample was sent for formal pathological analysis, while geographically discrete (0.7-1 cm3) tumour fragments (n=2-3) were used for tumour cell and TIL isolation after mechanical and enzymatic digestion (**Supplementary Fig. 1**). Fresh tumour fragments were minced under sterile conditions and dissociated into single-cell suspensions using the human Tumour Dissociation Kit and the gentleMAC dissociator (Miltenyi, Bergisch Gladbach, Germany). An aliquot of the cell suspension was analysed by flow cytometry (FC) and the remaining cells were magnetically labelled with non-tumour cell depletion cocktail A from the human Tumour Cell Isolation Kit (Miltenyi). Negative and positive fractions were sorted using an LS Columns (Miltenyi) and separately cryopreserved until further analysis. The positive fraction containing non-tumour cells was used for TIL isolation. Negative fraction enriched in tumour cells was used as autologous tumour targets for TIL reactivity testing.

Flow cytometry analysis

Tumour single-cell suspensions were incubated with Zombie NIR (ZN) Fixable dye (Biolegend, San Diego, CA, USA) and were, subsequently, stained with the following fluorochrome conjugated monoclonal antibodies (mAbs), as indicated: EPCAM-FITC (9C4), CD45-PECy7 (HI30), CD3-PercPCy55 (SK7), CD4-BV421 (RPA-T4), CD8-BV510 (SK1), PD-1-PE (EH12.2H7) and CD137-APC (4B4-1) (Biolegend) in FACS buffer [phosphate buffered saline (PBS) without Ca2+/Mg2+, heat-inactivated foetal calf serum (0.5%, SIGMA, San Luis, Missouri, USA), EDTA (2 mM, GIBCO, Dublin, Ireland) containing Beriglobin P (10 µg/ml, CSL Behring GmbH, Marburg, Germany) to block Fc receptors. Cells were acquired in a FACSCanto-II cytometer (BD Biosciences, Franklin Lakes, NJ, USA) and analysed using FlowJo software (BD Biosciences).

TIL isolation and expansion

Non-tumour cell-enriched fractions were thawed and rested overnight in T-cell media [1:1 mix of RPMI-1640-glutamax (Gibco) and AIMV (Gibco), supplemented with 5% heat-inactivated human serum (SIGMA), 12.5 mM HEPES, 100 U/ml penicillin, 100 µg/ml streptomycin, and 10 µg/ml gentamicin (Gibco)]. The next day, cells were stained with fluorochrome conjugated mAbs against CD8 (RPAT-8 or SK1) and PD-1 [EH12.2H7], and 7-amino-actinomycin D (dead cell marker) (Biolegend) and were sorted into PD-1 negative (PD-1^{-}) and PD-1 high (PD-1^{hi}) CD8^{+} T cells using a FACSAria cell sorter (BD Biosciences). Isolated cells were separately expanded by REP in T-cell media containing soluble anti-CD3 mAb (OKT3) (30 ng/ml, Biolegend), human IL-2 (3,000 IU/ml, Proleukin, Prometheus Laboratories Inc.,

San Diego, CA, USA), and 3×10^7 irradiated peripheral blood leucocytes pooled from 3 different donors. After 12-15 days of expansion, T cells were cryopreserved until further analysis.

TIL reactivity assessment

Expanded CD8 TIL and autologous tumour-enriched cells were thawed and separately rested overnight in T-cell media without IL-2. The next day, TIL (5×10^4 cells/well) were co-cultured either alone or with target cells [autologous tumour-enriched cells or the H929 plasmacytoma cell line, as unrelated tumour cells] (10^5 cells/well) in ELIIP plates (Millipore, Burlington, Massachusetts, USA) coated with purified anti-human IFN-γ mAb (Mabtech, Stockholm, Sweden), in the presence or absence of HLA-I blocking mAb (W6/32, Bio-x-cell, West Lebanon, NH, USA). Thirty-six hours later, cells were lysed with water, washed with PBS-Tween-20 (0.1%) and the plate was developed using biotinylated anti-IFN-γ mAb (7-B6-1) (1h, RT), streptavidin-ALP (1h, RT) and BCIP/NBT substrate (20', RT) (Mabtech). Results were analysed using a CTL-ImmunoSpot S6 MicroAnalyser (Cellular Technology, Cleveland, OH, USA). Data are expressed as the number of spot-forming cells (SFC) per 5×10^4 cells. For each patient, the number of target-specific IFN-γ SFC was determined by calculating the difference between the number of spots generated in the absence (TILs alone) or presence of target cells (autologous tumour or unrelated tumour). Reactivities >30 specific IFN-γ SFC and twice the background were considered positive. When autologous tumour-enriched cells were available, the reactivity of TILs was confirmed by assessment of CD137 upregulation. Briefly, TILs (1×10^5 cells/well) were cultured, either alone or with target cells ($0.5\text{-}1\times10^5$ cells/well), in T-cell media. Twenty-four hours later, cells were incubated with Zombie NIR Fixable dye. Then, they were stained with anti-human CD45-PECy7 (HI30), CD3-PercPCy55 (SK7), CD8-APC (SK1) and CD137-PE (4B4-1) mAbs (Biolegend), in the presence of Beriglobin P, and analysed by FC. CD8 TILs were identified as blastic $CD45^+CD3^+CD8^+$ living cells. H929 cells were acquired in 2017 from ATCC (CRL-9068) and used to produce the working bank that was cryopreserved in N2L. Cells with 1–4 passages from the working bank were used in the experiments. H929 cells were certified as being Mycoplasma-free by using the MycoAlert Mycoplasma Detection Kit (Lonza, Basel, Switzerland).

Multiplex immunofluorescence (IF) staining and analysis

Four-micrometre sections from full FFPE blocks from human ovarian tumours and control tonsil tissue were used for both, the initial setup of staining conditions for each single primary mAb and the successive optimization of multiplex staining. In the first step, primary mAbs were established as single stains initially on human tonsil tissue and thereafter on human ovarian tumours. The multiplex assay development and validation is shown on **Supplementary Fig. 2 and 3**. The antibodies used were: CD4 (clone 4B12, ready-to-use, Agilent, catalogue number IS64930-2), CD8 (clone C8/144B, ready-to-use, Agilent, catalogue number GA62361-2), FOXP3 (clone 236A/E7, 1:300, Abcam, catalogue number ab20034), PD1 (clone NAT105, ready-to-use, Cell Marque, catalogue number 315M), CD137 (clone BBK-2, 1:80, ThermoFisher, catalogue number MA5-13736), cytokeratin (clone AE1/AE3, ready-to-use, Leica Biosystems, catalogue

number NCL-L-AE1/AE). In a second step, multiplex IF was established essentially as described earlier [1,2]. Sequential rounds of mAb staining within a multiplex panel may lead to random binding of Abs, imbalanced signals, incomplete staining through interference with previously applied tyramide signal amplification (TSA), disruption of epitopes, and removal of TSA fluorophores because of repetitive antigen-retrievals at high temperature [1,3,4]. Therefore, each single mAb was optimized individually for its optimal position in the sequence of multiplex staining to minimize interference with previous Ab-TSA complexes or by alteration of epitopes (**Supplementary Fig. 3**).

For multiplex IF, 4-μm sections were deparaffinised and antigen retrieval was performed using DAKO PT-Link heat induced antigen retrieval with low pH (pH6) or high pH (pH 9) target retrieval solution (DAKO). Each section was subjected to six successive rounds of Ab staining, each round consisting of protein blocking with PerkinElmer Antibody Diluent/Block, incubation with primary mAbs, biotinylated anti-mouse/rabbit secondary antibodies and Streptavidin-HRP (Dako, 50003), followed by TSA visualization with fluorophores Opal 520, Opal 540, Opal 570, Opal 620, Opal 650, and Opal 690 (Akoya Biosciences) diluted in 1X Plus Amplification Diluent (Akoya Biosciences). Then, nuclei were counterstained with spectral DAPI (Akoya Biosciences) and sections mounted with DAKO Faramount S-3025.

Respective immunostainings without primary antibodies were used as negative controls. At equal Ab-concentrations, TSA-based visualization is expected to yield a higher number of positive cells as compared to conventional immunofluorescence [3,4]. We therefore established a monoplex TSA-based visualization of primary Abs on control tonsil tissue and ovarian tumour tissue as the gold standard for lymphocyte antigen visualization. Then, we performed a comparison monoplex vs. multiplex for each Ab to validate the staining patterns in human tonsil and ovarian tumour (**Supplementary Fig. 3**). Based on this comparison, we established the signal through dilution of the primary Abs to obtain staining levels and cell frequencies comparable to conventional immunofluorescence staining. The dilution of the CD137 antibody was optimized to allow for the detection of cytoplasmic CD137 in a similar pattern described earlier [5]. In multiplex IF, to monitor false-positive results through incomplete Ab-TSA complex-stripping and false-negative results through antigen masking (by incubation with multiple primary Abs, umbrella-effect), single primary Ab stainings were run in parallel and the results were then compared with those found with multiplex stainings. Spillover effects were controlled for anti-CD4-Ab and anti-CD137-Ab stainings on tonsil tissue with different Opal fluorophores by signal detection in adjacent cell components/channels and thereafter for exposure time settings upon acquisition of multiplex-stained tissue sections.

Tissue imaging, spectral unmixing, and phenotyping.

Multiplexed slides were scanned on a Vectra-Polaris Automated Quantitative Pathology Imaging System (Akoya Biosciences) as described [1,3,4]. A total of 6,95 mm^2 of tumour area for each case of ovarian cancer was scanned and analysed. Briefly, a spectral library from spectral peaks emitted by each fluorophore from single stained slides was generated using InForm software (version 2.4.8, Akoya Biosciences) and used for spectral unmixing of multispectral images allowing for identification of all markers of interest, as shown in

Supplementary Fig. 2. Auto- fluorescence was determined on an unstained ovary tumour tissue used in this study cohort. Tissue and cell segmentations were performed using InForm. Tissue segmentation algorithms based on cytokeratin staining allowed for distinct identification of cancer islands (tumour epithelium), stroma areas, and regions of non-interest, as described in **Supplementary Fig. 2**. Cell subpopulations were phenotyped in 20 random fields, representing 6.95 mm^2 of tumour area, as: $CD8^+$, $CD4^+$, and $CD4^+FOXP3^+$ and subsequently all cell subsets were scored as $PD1^{+/-}$ and/or $CD137^{+/-}$. The percentage of each cell population was calculated from the total number of cells in each of the 20 random fields examined and the average of the calculated field percentages was estimated. Average percentage was used, instead of cell density (mm^2), to allow similar comparisons and data normalization with the results from flow cytometry studies, in which the results were given in percentage of total population/parental population. Cells negative for theses markers were classified as other cell types. The staining pattern used to consider CD137 as positive was described earlier [5]. All phenotyping and subsequent quantifications were performed blinded to the sample identity.

Somatic mutation estimation

The mutational load was estimated using the Trusight Tumour 170 panel (TST170) from Illumina (San Diego, CA, USA). DNA were isolated from FFPE sections. One cut section from each tissue sample was stained with Haematoxylin and Eosin (H&E) and assessed for sample quality. Each H&E-stained slide was reviewed by a pathologist to evaluate the adequacy of tumour representation, the quality of tissue preservation, and whether significant artefacts relating to fixation, processing, or prefixation tissue handling were present. In samples from patients 3 and 10, laser capture microdissection was performed to enrich specimens with tumour tissue, under direct microscopic visualization. 10 to 20 5-µm-thick sections were used for DNA isolation. DNA extraction, library preparation and sequencing (NextSeq 500 using high-output cartridge and v2 chemistry) were performed by Macrogen according to Illumina's instructions. Sequence alignment and variant calling were performed using the TST170 BaseSpace application that applies an optimized version of Pisces Variant Caller to call single nucleotide variants (SNVs) and indels. Variant annotation was performed using Annovar (dbSNP150, 1000G, gnomad and COSMIC). We evaluated mutations that can cause non-synonymous SNVs, namely: missense variants and missense_variant&splice_Region_variant. Positions with depth <100, variant call quality <40, NC (fraction of bases which were uncalled or with basecall quality below the minimum threshold) ≥ 0,03 (for SNVs), strandbias score >-80, and variant allele frequency (VAF) <0.05 were ignored. Germline polymorphism filtering was performed by filtering Variant Allele Frequency (VAF) between 0.4-0.6 and >0.8. Polymorphisms were excluded if their Minor Allele frequencies (MAF) in European non-finnish (ENF) population (the ethnic group of our patients) according to gnomAD were >0. Mutations with MAF (ENF) = 0 or not found in gnomad were also not found in the 1000G database. We also evaluated mutations that can cause INDEL, namely: (i) conservative_inframe_insertion; (ii) conservative_inframe_deletion; (iii) disruptive_inframe_deletion, (iv) disruptive_inframe_insertion, (v) frameshift_variant; (vi)

frameshift_variant&splice_donor_variant&splice_region_variant&intron_variant; and (vii) frameshift_variant&splice_region_variant. To analyse these variants, filters similar to those used with SNVs were applied except NC threshold, which was not requested. Of the 2068 variants identified (**Supplementary Table S3**), 29 met the criteria for being somatic SNVs (**Supplementary Table S4 and S5**) and 25 for being somatic indels (**Supplementary Table S6, S7 and S8**). SNVs and INDELs were carefully checked by manual inspection of the sequenced reads using the Integrative Genome Browser (IGV). All putative SNVs were "truly SNVs" (highly confident SNVs), whereas 16/24 (60%) indels were considered as "Questionable INDELs" that may be false positives. The reasons for the questionability are summarized in **Supplementary Table S7**. Multiple genomic alterations in different patients were visualized using the oncoPrint function (R package ComplexHeatmap).

Analysis of mutations in genes from the DNA damage repair (DDR) system

Twenty-eight genes out of about 120 genes involved in direct DDR[6] and contained in the TST170 panel were examined (**Supplementary Tables S9 to S11**). All types of variants were considered, except for synonymous variants. Germline variants that may alter the DDR system were also accounted. A total of 53 variants were detected (5.3±1.9) affecting 18 genes (**Supplementary Table S9**). To identify disturbing mutations affecting these genes, the degree of certainty of pathogenicity was assessed in different database (Varsome, Clinvar, Cosmic, MutationTaster, PolyPhen-2 and CADD). Variants were considered "clinically relevant" if they were described as "Pathogenic" or " Likely Pathogenic" by Varsome and/or Clinvar and also as deleterious mutations by at least 2 of the following databases: MutationTaster, PolyPhen-2 and CADD (**Supplementary Table S9**).

NanoString-based gene expression profiling

After histopathological evaluation to certify the tumour content, five 5-µm-thick unstained FFPE sections were obtained from each tumour block. Samples were deparaffinised with xylene and digested with proteinase K and total RNA was extracted with the RNeasy FFPE kit (QIAGEN, Hilden, Germany) following the manufacturer's protocol. nCounter PanCancer Immune Profiling panel analysis (Nanostring, Seattle, Washington, USA) was performed by IMIBIC (Cordoba, Spain) following the manufacturer's instructions. Analyses of expression were performed using nSolver Analysis Software (v 4.0), with normalization utilizing positive and negative control probes, as well as the most stable housekeeping (HK) genes across samples. p-values <0.05 were considered to identify differentially expressed genes (DEG). Volcano-plots and unbiased clustering of DEG were generated using R (v3.5.3). For analysis of immune signatures, after HK normalization, a log10 transformation was applied, and the signature score was calculated by averaging the expression level of those genes included in (i) IFN-γ signature (6-gene: CXCL10, CXCL9, HLA-DRA, IDO1, IFNG, STAT1), (ii) a modified Expanded immune signature [including 15 genes (CCL5, CD2, CD3D, CD3E, CXCL10, CXCL13, CXCR6, GZMB, GZMK, HLA-DRA, HLA-E, IDO1, IL2RG, LAG3, STAT1) of the original 18 genes, and (iii) a modified T-cell inflamed

signature [including 16 genes (CCL5, CD27, CD274, CD276, CD8A, CMKLR1, CXCL9, CXCR6, HLA-DQA1, HLA-E, IDO1, LAG3, PDCD1LG2, PSMB10, STAT1 and TIGIT) of the original 18 genes[7]. Three (CIITA, NKG7, TAGAP) and two (NKG7 and HLA-DRB1) genes were not included in the Expanded immune signature and the T-cell inflamed signature, respectively, because they were absent in the PanCancer Immune Profiling Panel.

Statistical analysis

GraphPad Prism 6 software was used for graphic representation and most statistical analysis. Data from multiplexing IF were analysed using STATXact (Cytel Studio version 11.0.0). We used the Friedman test to identify differences among the 10 images of the same patient. If no statistically significant differences were found, then the images were considered homogeneous and the average frequency of each cell subset studied was calculated. To compare between reactive and non-reactive group we used non-parametric Mann-Whitney test, two-tailed (FC, TIL reactivity, mutational load, GEP score) or one-tailed (multiplexing IF) with 95% confidence interval. Detailed information is included in each figure legend.

References cited in Supplementary Methods

1 Gorris, M.A.J., Halilovic, A., Rabold, K., van Duffelen, A., Wickramasinghe, I.N., Verweij, D. *et al.* Eight-Color Multiplex Immunohistochemistry for Simultaneous Detection of Multiple Immune Checkpoint Molecules within the Tumor Microenvironment. *J Immunol* **200**, 347–354 (2018).

2 Martinez-Valbuena, I., Valenti-Azcarate, R., Amat-Villegas, I., Riverol, M., Marcilla, I., de Andrea, C.E. *et al.* Amylin as a potential link between type 2 diabetes and alzheimer disease. *Ann Neurol* **86**, 539–551 (2019).

3 Griss, J., Bauer, W., Wagner, C., Simon, M., Chen, M., Grabmeier-Pfistershammer, K. *et al.* B cells sustain inflammation and predict response to immune checkpoint blockade in human melanoma. *Nat Commun* **10**, doi:10.1038/s41467-019-12160-2 (2019).

4 Parra, E.R., Jiang, M., Solis, L., Mino, B., Laberiano, C., Hernandez, S. *et al.* Procedural requirements and recommendations for multiplex immunofluorescence tyramide signal amplification assays to support translational oncology studies. *Cancers (Basel)* **12**, doi:10.3390/cancers12020255 (2020).

5 Alfaro, C., Echeveste, J.I., Rodriguez-Ruiz, M.E., Solorzano, J.L., Perez-Gracia, J.L., Idoate, M.A. *et al.* Functional expression of CD137 (4-1BB) on T helper follicular cells. *Oncoimmunology* **4**, e1054597 (2015).

6 Chae, Y.K., Anker, J.F., Carneiro, B.A., Chandra, S., Kaplan, J., Kalyan, A. *et al.* Genomic landscape of DNA repair genes in cancer. *Oncotarget* **7**, 23312–23321 (2016).

7 Ayers, M., Lunceford, J., Nebozhyn, M., Murphy, E., Loboda, A., Kaufman, D.R. *et al.* IFN-γ-related mRNA profile predicts clinical response to PD-1 blockade. *J Clin Invest* **127**, 2930–2940 (2017).

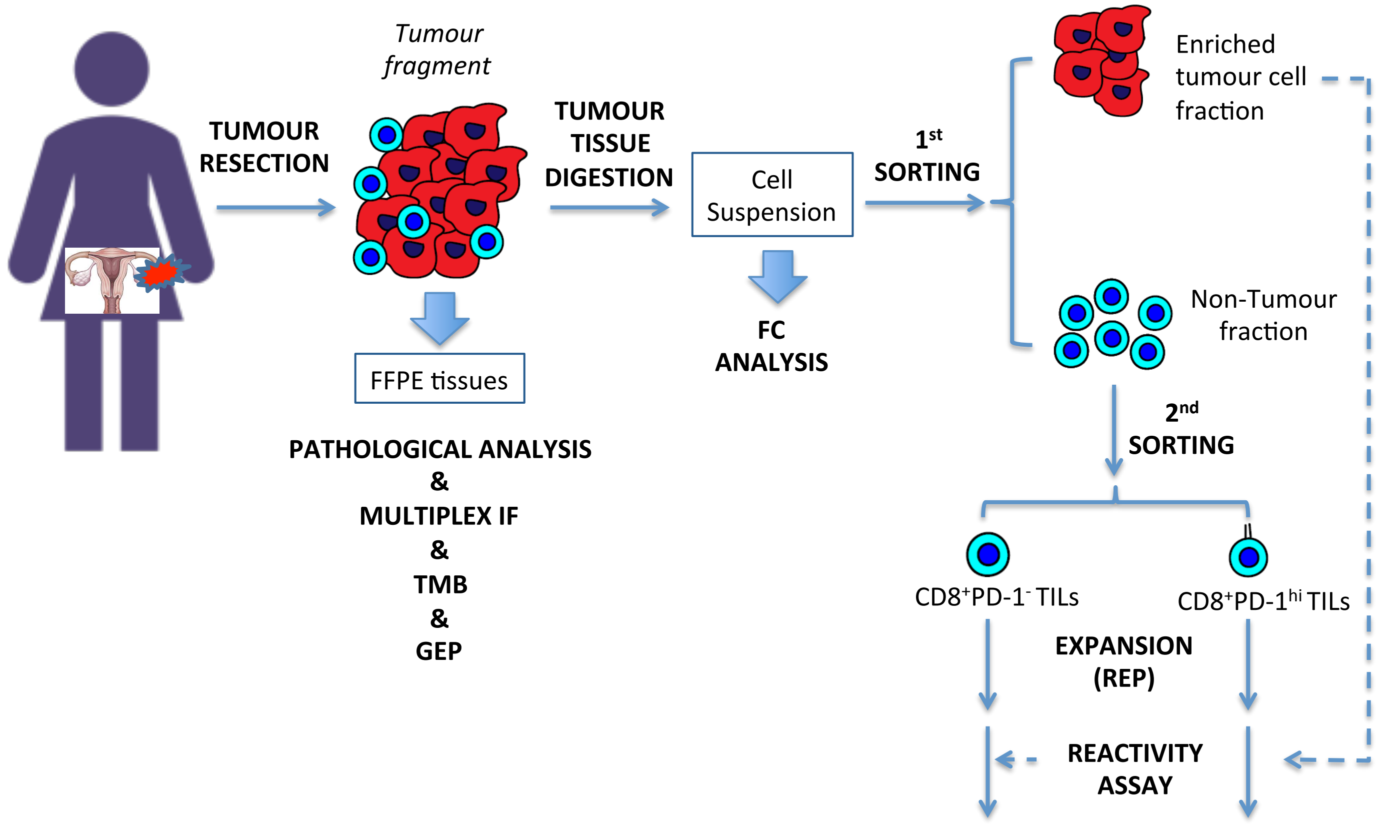

Supplementary Figure 1. Flow Chart. Ovarian cancer patients underwent debulking cytoreductive surgery. A representative sample of tumour was used to prepare formalin-fixed paraffin-embedded (FFPE) tissue samples for formal pathological analysis, while 0.7-1 cm^3 tumour fragments were minced and dissociated into single-cell suspensions. An aliquot of the tumour cell suspension was analysed by flow cytometry (FC) and the remaining cells were magnetically sorted into tumour cells and non-tumour cells using the human Tumour Cell Isolation Kit (Miltenyi) (1st sorting) and cryopreserved at N_2L. The tumour-enriched fraction was used as autologous tumour targets for TIL reactivity testing, whereas the non-tumour fraction was used for TIL isolation. Both fractions were separately cryopreserved until further use. After thawing and resting overnight, non-tumour cells were stained with mAbs against CD8 and PD1 and then $CD8^+PD1^-$ and $CD8^+PD1^{hi}$ TILs were isolated by Fluorescence-activated cell sorting (2nd sorting). Isolated subsets were separately expanded following the "rapid expansion protocol" (REP). After 12-15 days of expansion, T cells were cultured with autologous tumour cells and antitumor reactivity was measured by IFNγ ELISPOT. FFPE samples were retrospectively used for multiplex quantitative immunofluorescence (IF), tumour mutational burden (TMB) and gene expression profile (GEP) analyses.

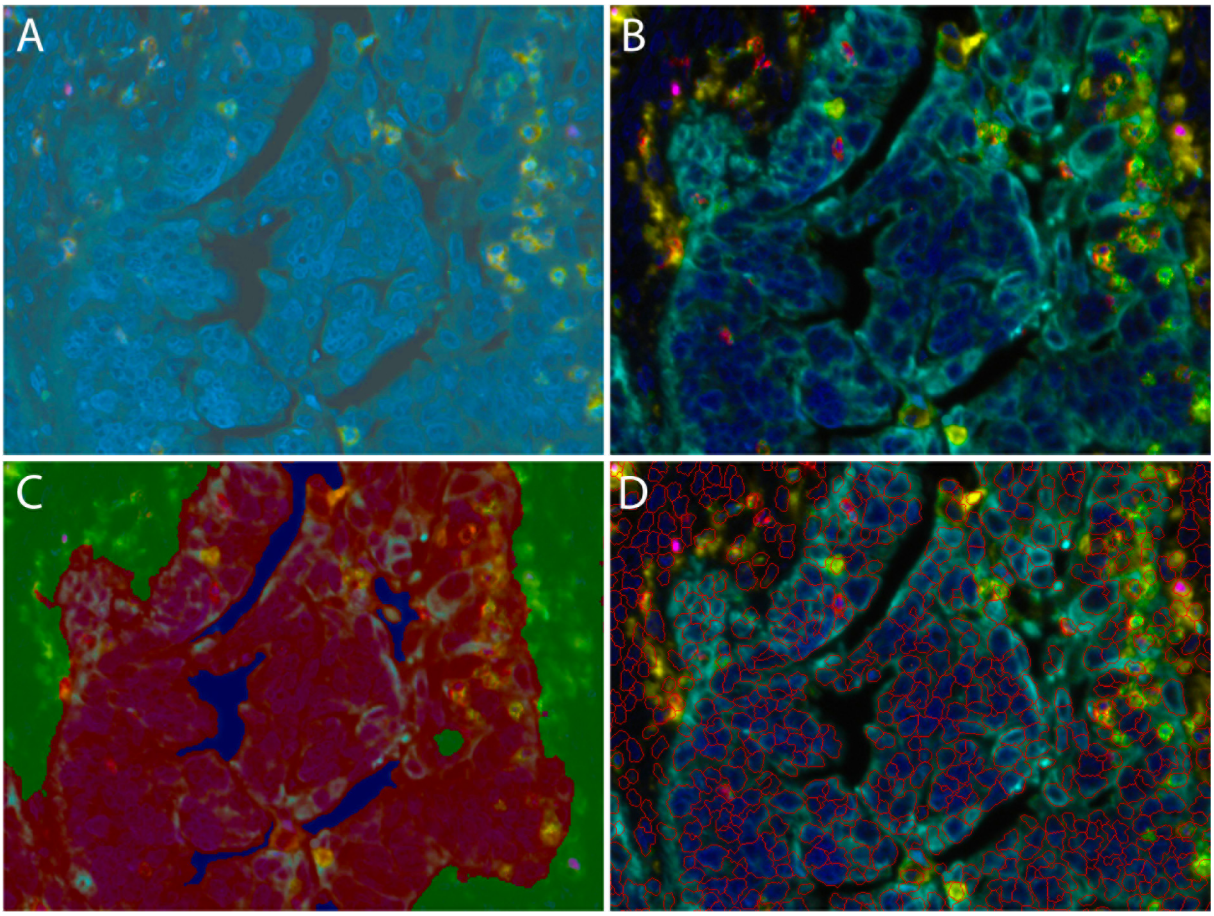

Supplementary Figure 2. Image analysis using Inform software. (A) Formalin-fixed, paraffin-embedded (FFPE) tissues were imaged using the Akoya Biosciences' Vectra-Polaris imaging platform and the resulting .im3 image files were spectrally unmixed using Akoya Biosciences' Inform software (version 2.4.8). (B) Spectrally unmixed composite image of papillary serous cystadenocarcinoma of ovary (patient P05) stained against CD137 (clone TNFRSF9, Opal 520, orange), PD1 (clone NAT-105, Opal 540, green), CD4 (clone 4B12, Opal 650, yellow), CD8 (clone C8/144B, Opal 570, red), FoxP3 (clone 236A/E7, Opal 570, magenta), panCK (clone AE1/AE3, Opal 690, cyan), and DAPI (Dark Blue). (C) Tissue segmentation algorithms based on panCK staining allowed for distinct identification of cancer islands (red), stroma areas (green), and regions of non-interest (blue). (D) Cell segmentation algorithms based on the expression of different markers, such as nucleus (DAPI), cytoplasm, or membrane, were used to accurately identify each cell.

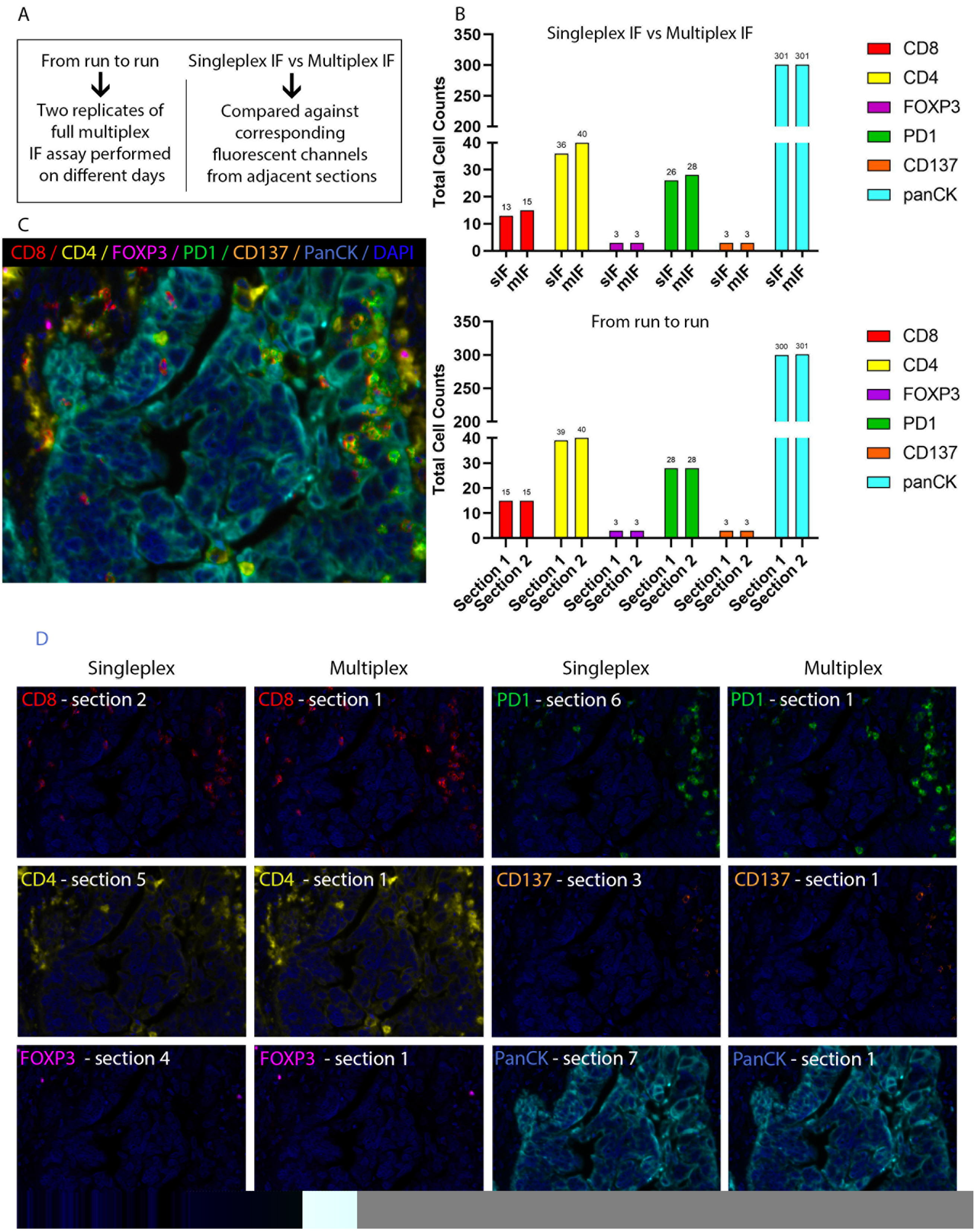

Supplementary Figure 3. Validation results. (A) Validation workflow. (B) Total number of cells for each marker, comparing singleplex immunofluorescence (IF) against the corresponding fluorescent channel from multiplex IF on sequential FFPE tumour sections and replicates of full mIF assays performed on different days. (C) Unmixed 7-color multispectral image of a case of papillary serous cystadenocarcinoma of the ovary (patient P05) used for the validation studies. (D) Multiplex IF images were compared against the corresponding fluorescent channel from singleplex IF images from sequential sections (mIF on section 1; sIF on sections 2-7).

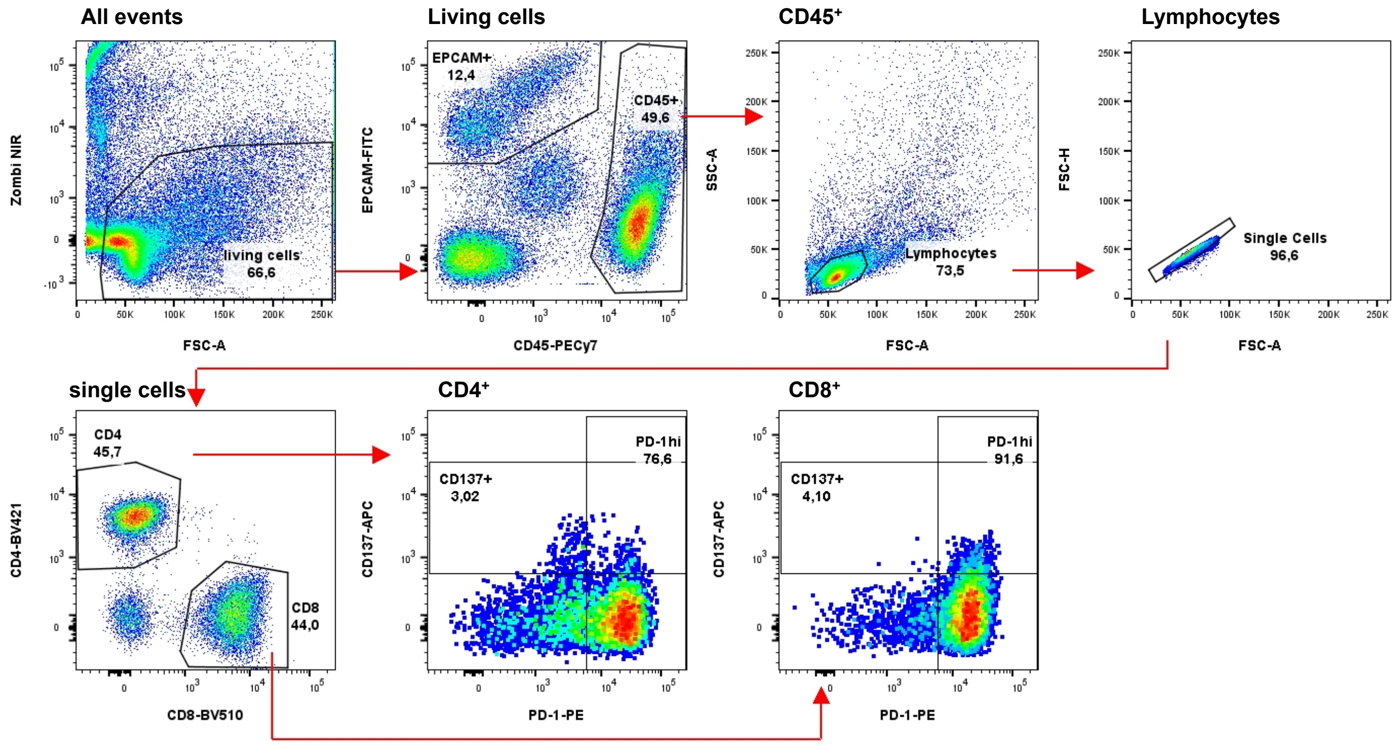

Supplementary Figure 4. Gating strategy and example of data from patient P05. Dead cells were removed from the analysis using Zombi NIR fixable dead cell stain (1st plot). Next, cells were gated based on CD45 and EPCAM expression (2nd plot). Lymphocytes were gated from CD45$^+$ cells based on FSC and SSC (3rd plot). Doublets were removed from lymphocytes using FSC-A and FSC-H (4th plot). The 5th plot shows CD4$^+$ and CD8$^+$ cell gates and the 6th and 7th plots depict the expression of PD-1 and CD137 in CD4 and CD8 TILs.

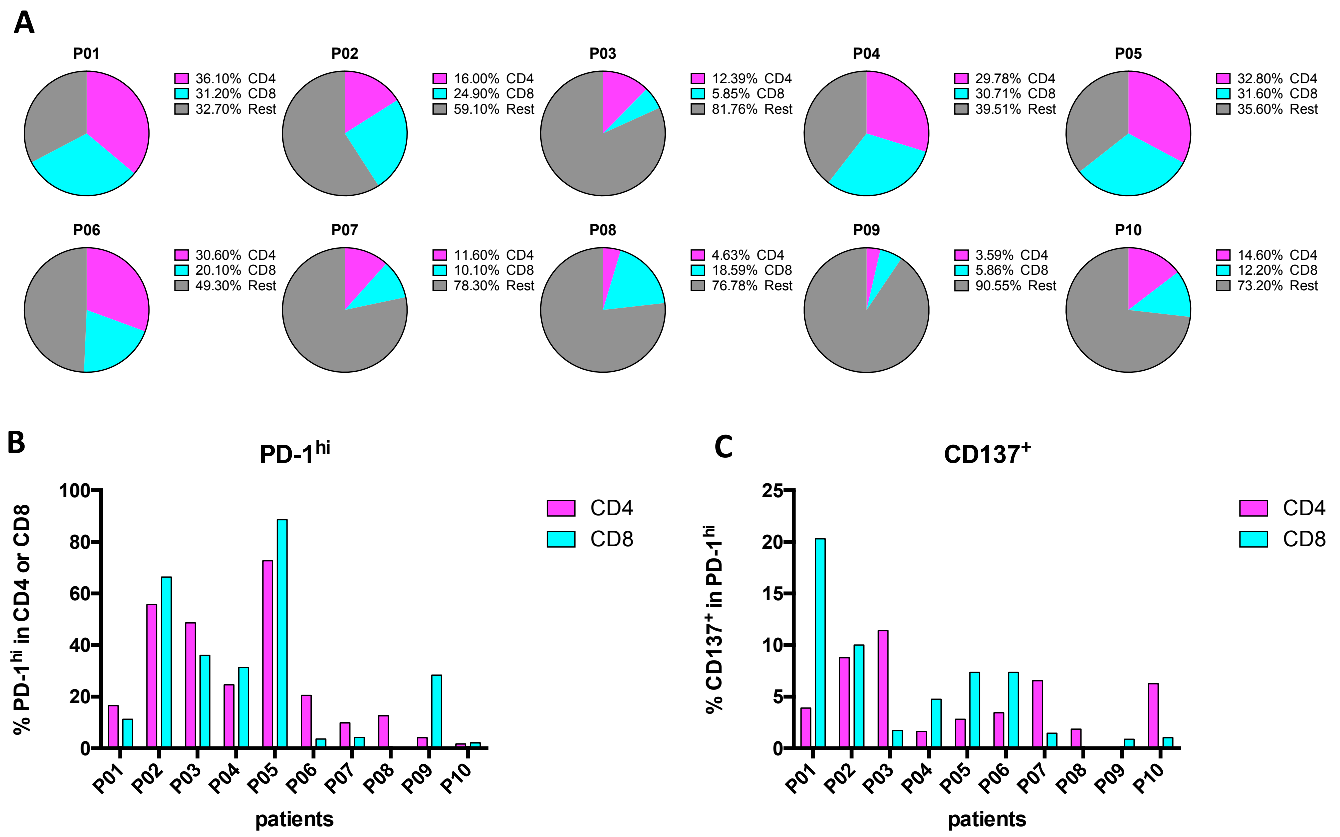

Supplementary Figure 5. Percentage and phenotypic traits of CD4 and CD8 TILs in the fresh tumour. Tumour single-cell suspensions were analysed by FC as detailed in Material and methods. The gating strategy is described in Supplementary Fig. 2. (**A**) Percentage of $CD4^+$ (pink) and $CD8^+$ (clear blue) cells in $CD45^+$ cells. In grey, the remaining $CD45^+$ cells. (**B**) Percentage of PD-1^{hi} cells in $CD4^+$ and $CD8^+$ cells. (**C**) Percentage of $CD137^+$ cells in PD-$1^{hi}CD4^+$ or PD-$1^{hi}CD8^+$ subsets.

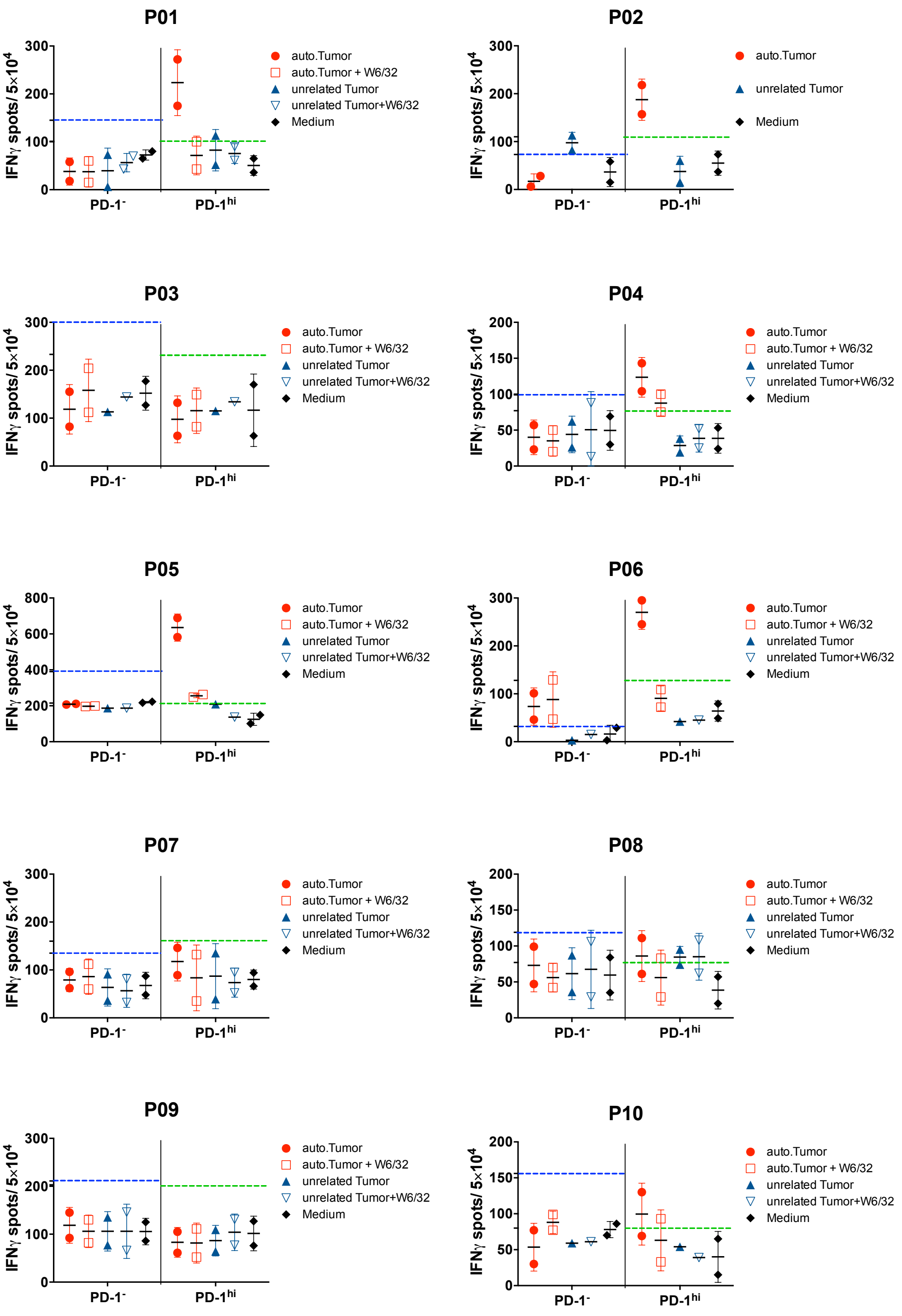

Supplementary Figure 6. The ability of PD-1-selected cells to render TR TIL products varied among patients. Cells expanded from the PD-1^{-} or the PD-1^{hi} CD8 TIL subset were co-cultured with the respective autologous tumour cells, or with unrelated tumour cells (H929), in the presence or absence of HLA-I blocking antibody (W6/32) and tumour recognition was assessed by measuring IFN-γ release by ELISPOT. The graph shows the number of spots per 5×10^4 cells (mean±SD). Blue and green horizontal dotted lines show the background level [twice the number of spots generated in the absence of targets cells (Medium)] for PD-1^{-}- and PD-1^{hi}-derived cells, respectively. A TIL product was considered TR if the two replicates were above the background level and the difference between the average IFN-γ spot numbers and the background was >30.

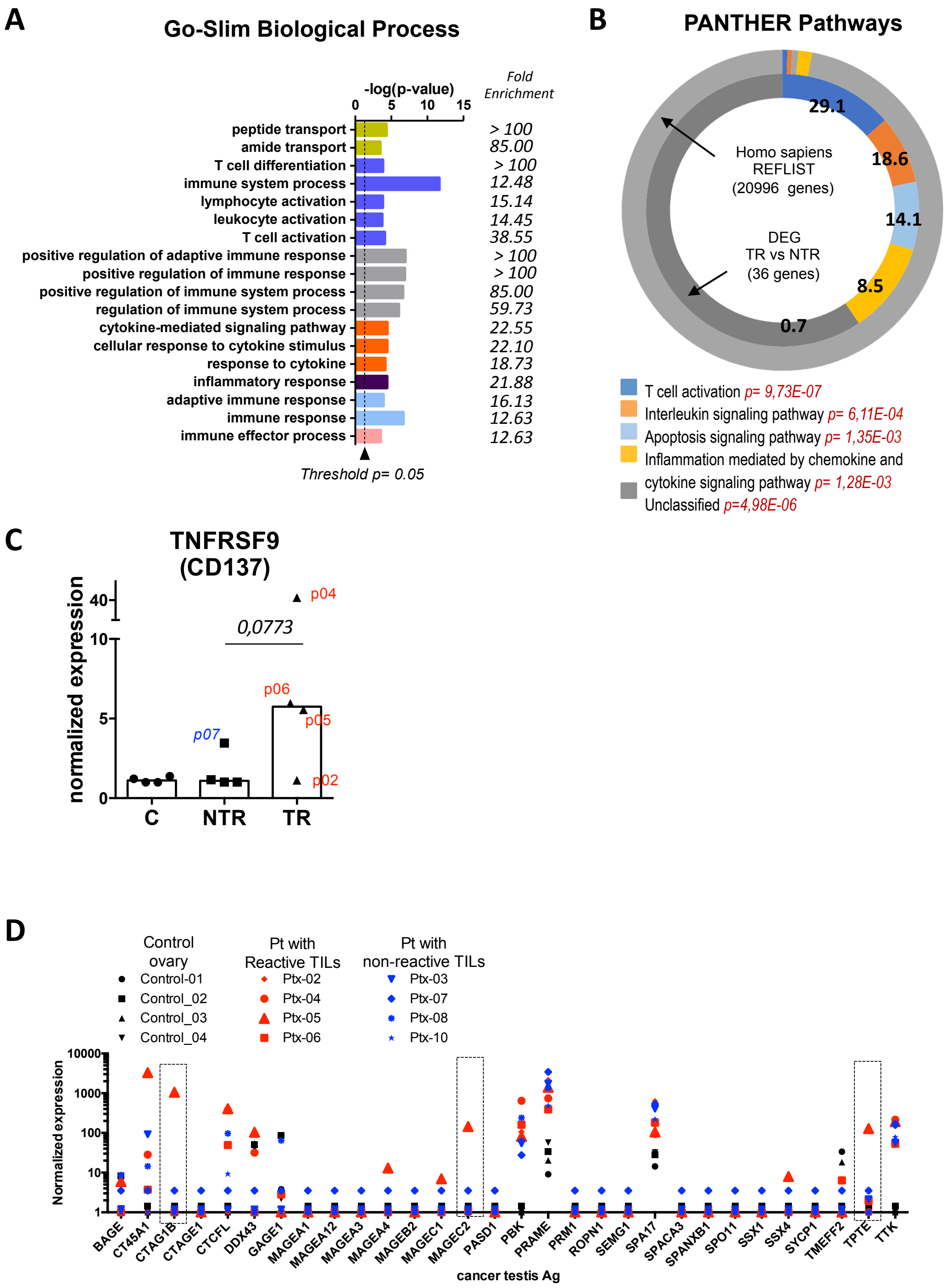

Supplementary Figure 7. Transcriptome traits of patients with TR TILs. (**A and B**) Transcript gene functional analysis was conducted using the PANTHER gene ontology (GO) database. Graphs show data from overrepresentation Tests using Bonferroni correction. (**A**) Overrepresented GO biological processes. Biological process has been grouped by category. Numbers on the right indicate the fold enrichment. (**B**) Overrepresented GO biological pathway. External circle: Homo sapiens REFLIST (20996 genes). Internal circle: DEG TR vs. NTR (36 genes). The length of arc indicates the percentage of genes within the pathway. Bold numbers over each arc indicate the Fold enrichment in each category (observed/expected count). (**C**) Expression of the TNFRSF9 (CD137) transcript in ovary samples from TR and NTR patients and control subjects. (**D**) Expression of Cancer testis antigen (CTA) transcripts included in PanCancer Immune Profiling Panel across ovarian tumours and control ovaries from the study. Each point represents a subject. Those CTAs specifically expressed by the patient P05 are highlighted.

Supplementary Table 1. Patient's clinical and cytological characteristics

Patient	Histopathological subtype	Stage (FIGO)(1)	**Age**	**Previous treatment**	Surgical Endpoint	Evolution after surgery
P01	High Grade Papillary-Serous Cystoadenocarcinoma	IIIC	63	None	Suboptimal cytoreduction	Progression after adjuvant ChT. FPI: 12 months
P02	Mucinous Cystoadenocarcinoma	IIA	45	None	Complete cytoreduction	Non progression
P03	Endometrioyd Carcinoma	IC	49	None	Complete cytoreduction	Non progression
P04	High Grade Papillary-Serous Cystoadenocarcinoma	IA	63	None	Complete cytoreduction	Non progression
P05	High Grade Papillary-Serous Cystoadenocarcinoma	IIIB	85	None	Suboptimal cytoreduction	Non progression
P06	High Grade Papillary-Serous Cystoadenocarcinoma	IIB	74	None	Optimal Cytoreduction	Non progression
P07	Endometrioyd Carcinoma	IIIC	57	None	Complete cytoreduction	Non progression
P08	High Grade Papillary-Serous Cystoadenocarcinoma	IIIB	52	None	Optimal Cytoreduction	Progression after adjuvant ChT. FPI: 17 months
P09	High Grade Papillary-Serous Cystoadenocarcinoma	IIIC	48	None	Complete cytoreduction	Progression after adjuvant ChT. FPI: 26 months
P10	High Grade Papillary-Serous Cystoadenocarcinoma	IIIC	69	None	Optimal Cytoreduction	Progression after adjuvant ChT. FP1: 4 months

(1) FIGO 7th Edition. ChT: Chemotherapy. FPI: free platinum interval after last platinum adjuvant dose.

Supplementary Table 2. Number of cells isolated from fresh tumours and fold expansion after REP.

	Isolated cell numbers(1)		Fold Expansion(3)		
Patient	$PD1^-$	$PD1^{hi}$	Day(2)	$PD1^-$	$PD1^{hi}$
P01	102 200	21 200	13	959	144
P02	15 300	127 142	13	167	306
P03	15 879	1 759	13	567	441
P04	8 058	11 204	14	345	455
P05	13 000	39 880	15	2 677	853
P06	3 919	2 665	14	561	454
P07	176 422	43 996	12	485	743
P08	43 599	20 649	12	344	489
P09	17 286	31 355	12	810	399
P10	10 000	13 000	12	262	198

(1) Number of isolated cells, according to FACSAria sorter. This number coincides with the number of cells in the starting REP culture. (2) Last day of REP culture. (3) Fold-expansion was calculated by dividing the number of cells at the end of the expansion phase by the number of cells in the starting culture. $PD\text{-}1^-$ and $PD\text{-}1^{hi}$ cells expanded efficiently and no differences were found in fold-expansion between both subsets (Mean±SEM: 450±91.55 and 448±69.15, respectively)
